# Reprogramming of the m^6^A Epitranscriptome Drives Triptolide-Induced Reproductive Toxicity in HTR-8/SVneo Cells

**DOI:** 10.3390/toxics14040334

**Published:** 2026-04-16

**Authors:** Xinru Liu, Yunli Wu, Jin Tian, Jiaxin Wen, Yuan Shi, Lili Wang, An Zhu, Zekai Wu

**Affiliations:** 1 Key Laboratory of Gastrointestinal Cancer, Fujian Medical University, Ministry of Education, Fuzhou 350122, China; xinru_liu@fjmu.edu.cn (X.L.); wuyunli422@163.com (Y.W.); tianjin1128@fjmu.edu.cn (J.T.); jiaxin-wen@fjmu.edu.cn (J.W.); 2 State Key Laboratory of Mariculture Breeding, College of Marine Sciences, Fujian Agriculture and Forestry University, Fuzhou 350002, China; shiyuan@fafu.edu.cn; 3 Department of Radiology, Fujian Medical University Union Hospital, Fuzhou 350001, China; fjwll1@fjmu.edu.cn; 4 Fujian Key Laboratory of Tumor Microbiology, Department of Medical Microbiology, School of Basic Medical Sciences, Fujian Medical University, Fuzhou 350122, China

**Keywords:** triptolide (TPL), epitranscriptomics, trophoblast cells, reproductive toxicity, N^6^-methyladenosine (m^6^A)

## Abstract

Triptolide (TPL), the core active component of the traditional Chinese medicinal herb *Tripterygium wilfordii* Hook F (TwHF), possesses a wide spectrum of pharmacological activities, including anti-inflammatory, neuroprotective, immunosuppressive, and anti-tumor activities. However, its clinical application is severely limited by significant reproductive toxicity, the mechanism of which remains poorly understood. Using an integrated analysis of MeRIP-seq and mRNA-seq data, coupled with experimental validation in HTR-8/SVneo cells, we systematically elucidated the molecular mechanism by which TPL induces trophoblast cell injury. Our findings revealed that TPL significantly altered intracellular N^6^-methyladenosine (m^6^A) modification and gene expression profiles, with 1774 genes displaying hypomethylation concurrent with mRNA upregulation. According to the functional enrichment analysis, these genes showed significant enrichment in several key pathways associated with reproduction, including autophagy, DNA damage response, mitochondrial outer membrane, and positive regulation of apoptotic process. Molecular docking further demonstrated direct and stable binding of TPL to key m^6^A regulators, leading to specific demethylation of targets including *E2F1* and *PPP1CC*. This study uncovers a novel post-transcriptional mechanism where TPL disrupts m^6^A modification, thereby perturbing essential trophoblast functions and driving reproductive toxicity.

## 1. Introduction

*Tripterygium wilfordii* Hook F (TwHF) is a traditional Chinese medicinal herb that is clinically used for dispelling wind and dampness, which alleviates symptoms commonly associated with rheumatic immune diseases, such as joint swelling, pain and limited mobility, as well as for activating blood circulation and unblocking collaterals, killing parasites and removing toxins, and reducing swelling and relieving pain [1]. It has a definite curative effect in the treatment of rheumatic immune diseases and has obvious advantages, especially in adjuvant therapy and combination medication. Studies have shown that, in patients with rheumatoid arthritis, the improvement effect of combined TwHF adjuvant therapy on disease activity is significantly better than that of conventional therapy alone [2]. Another study based on 11 randomized controlled trials involving 1026 patients with rheumatic immune diseases indicated that the combination of TwHF tablets and Western medicine can effectively reduce the risk of disease recurrence in patients [3]. A meta-analysis including 8 randomized controlled trials with a total of 583 subjects also confirmed that compared with the single use of glucocorticoids, the combination of TwHF tablets can significantly improve the overall remission rate of patients with lupus nephritis [4]. However, the reproductive and developmental toxicity of TwHF cannot be ignored. Existing in vitro studies have shown that TwHF extracts at concentrations of 50 and 100 μg/mL exhibit significant embryotoxicity in mouse embryos on days 9 and 10. These extracts induce various morphological abnormalities, including branchial apparatus malformation, cranial neural tube opening, and limb bud loss. They also lead to reduced somite number, decreased axial length, and diminished yolk sac surface area, accompanied by extensive cell death. In addition, abnormal embryos also exhibit impaired blood circulation in the visceral yolk sac and ultrastructural damage, which further affects their normal development [5]. Triptolide (TPL), the main active ingredient of TwHF, is a diterpenoid triepoxide lactone compound with various pharmacological activities such as anti-tumor and anti-inflammatory effects [6,7]. Notably, TPL can cross the blood–brain barrier and promote neural repair after cerebral ischemia–reperfusion injury, thereby exerting neuroprotective effects [8]. Additionally, its broad immunosuppressive activity has significant effectiveness in inhibiting the development of transplant vasculopathy [9]. Some studies suggest that the reproductive toxicity of TwHF may be related to its various main components, and TPL, as the main active ingredient, may play an important role in inducing reproductive toxicity [10]. However, current understanding of the precise molecular mechanisms underlying TPL-induced reproductive toxicity remains limited, which to some extent restricts the safe clinical application of TwHF.

The development of epitranscriptomics has provided a cutting-edge strategy for dissecting the mechanisms of drug toxicity. This field focuses on RNA modifications and their downstream regulatory effects, representing a new layer of genetic information regulation encompassing diverse modification types such as m^6^A, pseudouridine, and 2′-O-methylation [11,12]. As one of the most extensively studied mRNA modifications in eukaryotes, m^6^A modification was initially detected in diverse organisms in the 1970s [13,14]. As a type of epitranscriptomic modification, its dynamic and reversible regulation is mediated by three categories of proteins. “Writers” (e.g., METTL5/VIRMA/WTAP) add methyl groups to adenines (A) on RNA molecules to form m^6^A. “Erasers” (e.g., ALKBH5/FTO) specifically “erase” pre-existing m^6^A modifications. “Readers” (e.g., HNRNPC/IGF2BP1/YTHDC1) specifically recognize m^6^A sites and mediate downstream effects, ultimately determining RNA fate and function [15]. m^6^A modification exhibits typical site preference, being mainly enriched near the stop codon and the 3′ untranslated region of mRNAs, and most of these modifications follow the RRACH consensus motif (where R = G or A, and H = A, C, or U) [13]. It influences gene expression by regulating RNA processing, translation, and degradation during cell differentiation, embryonic development, and stress responses [16,17].

Trophoblast cells are composed of multiple functionally distinct subpopulations that play critical roles in placental development and uterine wall adaptation during pregnancy. The normal composition of these subpopulations is essential for proper placental development, and any functional abnormalities can lead to pregnancy disorders such as fetal developmental abnormalities [18]. Notably, a clinical case report described a male infant born with occipital encephalocele and cerebellar hypoplasia, whose mother had been treated with TwHF for rheumatoid arthritis during the first trimester [19]. This case suggests that first-trimester exposure to TwHF may be associated with severe fetal neurodevelopmental abnormalities, which often arise from trophoblast dysfunction. Given that TPL is the main active component of TwHF, this study selected the immortalized human extravillous trophoblast cell line HTR-8/SVneo as a research model, combined with epitranscriptomics, to investigate the reproductive toxicity mechanism of TPL [6]. Specifically, the toxic effects of TPL and its core effective concentration were determined using the Cell Counting Kit-8 (CCK-8) assay. Also, flow cytometry was performed to detect the effect of TPL on the apoptosis rate. Subsequently, mRNA-seq and MeRIP-seq analyses were performed at this concentration to screen TPL-induced differentially expressed genes (DEGs) and differentially m^6^A-modified genes, and to explore pathways related to trophoblast cell function and core m^6^A regulators. Molecular docking was used to simulate the interaction between TPL and key m^6^A regulators, and the gene expression levels of these regulators were detected by real-time quantitative PCR (RT-qPCR). Ultimately, this study aims to reveal whether TPL impairs trophoblast cell function by interfering with m^6^A modification, thereby providing new insights into the regulatory mechanisms underlying its reproductive toxicity.

## 2. Materials and Methods

### 2.1. Chemical Reagents

TPL (C_20_H_24_O_6_) was purchased from Must Bio-Technology Co., Ltd. (Chengdu, China). Its chemical structure was verified by 400 MHz^1^H nuclear magnetic resonance (NMR) spectroscopy (Figure S1A,B). The purity of TPL was determined to be 99.87% by high-performance liquid chromatography (HPLC), which met the experimental requirements (Figure S1C).

### 2.2. PharmMapper Analysis

The mol2 file format of TPL was prepared by retrieving its 2D structure from the PubChem database and processing it with Chem3D Ultra v20.0.0.41 software [20]. The file was loaded into SYBYL-X v2.1.1 software for energy minimization optimization, and then uploaded to the PharmMapper database to predict potential targets. Proteins with top-ranking scores were selected as candidate targets according to the fitting score, and imported into the DAVID database (https://davidbioinformatics.nih.gov/) for Gene Ontology (GO) functional annotation and Kyoto Encyclopedia of Genes and Genomes (KEGG) pathway enrichment analysis, aiming to preliminarily explore the possible biological mechanisms of TPL.

### 2.3. Cell Culture and Treatment

The HTR-8/SVneo cell line was purchased from the American Type Culture Collection (Manassas, VA, USA) and maintained in DMEM complete medium supplemented with 10% fetal bovine serum. Cells were cultured at 37 °C in a humidified atmosphere with 5% CO_2_. Before the experiment, TPL was dissolved in sterile DMSO and prepared into working solutions at 5, 10, 15, and 20 nM. HTR-8/SVneo cells were treated with these solutions for 48 h, with 0.1% sterile DMSO serving as the vehicle control.

### 2.4. Cell Viability Assay

The CCK-8 assay was utilized to assess the effect of TPL on the viability of HTR-8/SVneo cells. The CCK-8 kit was purchased from Abbkine Biotechnology Co., Ltd. (Wuhan, China). First, HTR-8/SVneo cells were seeded at an appropriate density into 96-well plates and incubated for 24 h. The medium was then replaced with medium containing 0, 5, 10, 15, and 20 nM TPL, and the cells were cultured for another 48 h. After this treatment, the culture medium in the 96-well plates was removed, and 100 μL of CCK-8 working solution (DMEM medium: CCK-8 reagent = 9:1) was added to each well. After a 1 h incubation, the absorbance was recorded at 450 nm using a microplate reader, and cell viability was determined accordingly.

### 2.5. Apoptosis Detection

Annexin V-FITC/PI double staining was used to detect the apoptosis of HTR-8/SVneo cells, and the kit was purchased from Vazyme Biotech Co., Ltd. (Nanjing, China). The main steps were as follows: HTR-8/SVneo cells were seeded at an appropriate density into culture dishes and incubated for 24 h. Subsequently, TPL working solutions were added to the culture medium to achieve final concentrations of 0, 5, 10, 15, and 20 nM, respectively, and the cells were cultured for an additional 48 h. After treatment, the cells were harvested and processed for staining. Finally, blank tubes, single-staining tubes, and experimental sample tubes were analyzed using a FACSCanto^TM^ II flow cytometer (BD Biosciences, San Jose, CA, USA).

### 2.6. RNA Extraction

HTR-8/SVneo cells were seeded in six-well plates and cultured for 24 h, followed by treatment with 0, 5, 10, 15, and 20 nM TPL for 48 h. Cells were then washed twice with PBS, and total RNA was extracted using Trizol reagent (Invitrogen, Carlsbad, CA, USA). Genomic DNA was removed by DNase I treatment. The concentration and purity of the RNA samples were determined using a Nanodrop One C spectrophotometer (Thermo Fisher, Waltham, MA, USA) [21].

### 2.7. mRNA-Seq and MeRIP-Seq

The cytotoxicity assay results showed that the viability of HTR-8/SVneo cells in the 15 nM TPL treatment group was approximately 60%, and this concentration was used as the core working concentration for subsequent experiments. A total of 50 µg of qualified RNA was entrusted to Seqhealth (Wuhan, China) for mRNA-seq and m^6^A MeRIP-seq. The KC-Digital Stranded mRNA Library Prep Kit for Illumina (Seqhealth, Wuhan, China) was used for library construction to suppress duplication bias, and fragments of 200–500 bp in length were enriched and quantified. Biological replicates were set in the experiment, and three independent samples were included in each group to ensure data reliability. The sequencing strategy of this study is based on the core design framework of transcriptomics research on placental trophoblast cells, and systematically elucidates the potential molecular mechanisms of toxin-induced injury to placental trophoblast cells through omics technologies [22,23].

### 2.8. Sequencing Data Analysis

MeRIP-seq raw data were aligned to the hg19 reference genome sequences using HISAT2 [24]. The m^6^A peaks were extracted by exomePeak2 (Suzhou, China), and STREME was used for motif identification [25,26]. Differential mRNA expression profiles between TPL-treated and control groups were analyzed using StringTie (Baltimore, MD, USA) and DESeq2 [27,28]. DEGs were selected with |log_2_FC| ≥ log_2_(1.2) and *p*-value < 0.05. GO and KEGG enrichment analyses of DEGs were then performed using the DAVID database [29]. For key pathways, protein–protein interaction (PPI) networks of core genes and m^6^A regulators were constructed using Cytoscape v3.10.3 software [30,31]. Core target genes were screened by sorting according to degree values, and MeRIP-seq/mRNA-seq coverage signal plots for these genes were generated using Integrative Genomics Viewer (IGV) v2.19.6 software. The association between m^6^A modification and diseases was analyzed by RMDisease (www.xjtlu.edu.cn/biologicalsciences/rmd, accessed on 20 September 2025) [32]. The substrates of regulators were obtained from RM2Target (http://rm2target.canceromics.org/, accessed on 17 November 2025) [33].

### 2.9. Molecular Docking

The binding interaction between differentially expressed m^6^A regulators and TPL was analyzed using AutoDockTools v1.5.7 software. First, the target protein structures were obtained from the AlphaFold database and the PDB database, followed by pretreatment using AutoDockTools v1.5.7 software, including the removal of water molecules and heteroatoms, the addition of hydrogen atoms, and other operations. The processed target protein was used as the receptor, saved in PDBQT format. Next, the 2D structure of TPL was downloaded from the PubChem database, converted to mol2 format via Chem3D Ultra v20.0.0.41 software, and then subjected to energy minimization using SYBYL-X v2.1.1 software. Subsequently, the small molecule was hydrogenated using AutoDockTools v1.5.7 software, set as the ligand, followed by checking its torsional bonds and centers, and then exported in PDBQT format. Then, the PDBQT files of the target protein and the small molecule were opened separately, and the size of the docking box was set to ensure that the protein was enclosed within the box while the small-molecule ligand was located outside the box. Finally, molecular docking between the target protein and the small molecule was performed. A binding energy < −1.2 kcal/mol was used as the criterion, and values below this threshold indicated a stable interaction between the protein and the small molecule.

### 2.10. RT-qPCR Analysis

Total RNA was extracted using the Trizol method from HTR-8/SVneo cells treated with different concentrations of TPL for 48 h. Approximately 1 μg of RNA was reverse transcribed into cDNA using a reverse transcription premix kit. The RT-qPCR reaction system was 20 μL, containing 0.5 μL of forward and reverse primers each, 10 μL of 2 × SYBR Green qPCR Master Mix, 2 μL of cDNA, and 7 μL of ddH_2_O. GAPDH was used as the internal reference gene, and the primer sequences are listed in Table 1. The relative expression levels of target genes were calculated with the 2^−ΔΔCt^ method [34,35].

### 2.11. Statistical Analysis

Statistical analysis was performed using GraphPad Prism v8.0.2 software. Homogeneity of variance was tested by the Brown-Forsythe test, and differences among groups were compared using one-way analysis of variance (ANOVA). Data are presented as mean ± standard error of the mean (SEM), with *p* < 0.05 considered statistically significant.

## 3. Results

### 3.1. TPL Concentration-Dependently Reduced Cell Viability

**Figure 1:**
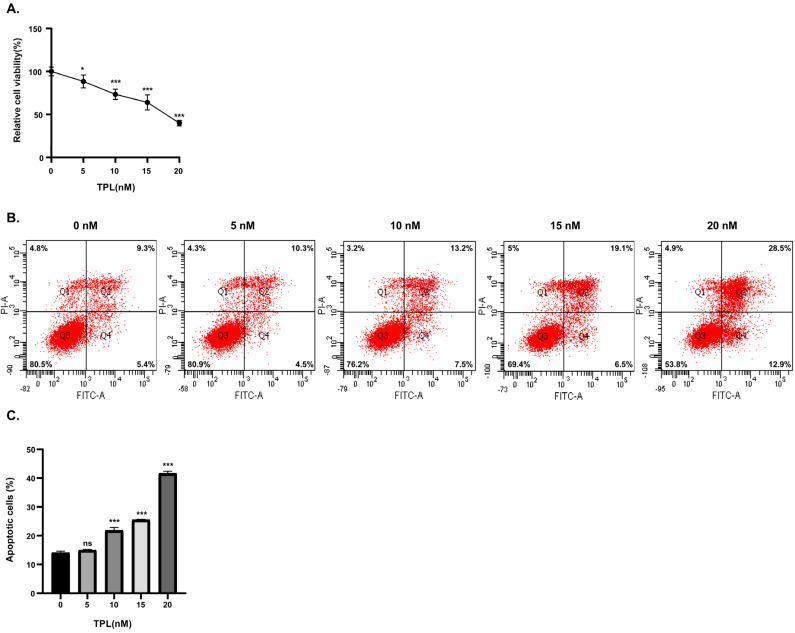
Results of cell viability and apoptosis assays in HTR-8/SVneo cells treated with TPL. (**A**) Cell viability of HTR-8/SVneo cells treated with TPL measured by CCK-8 assay; ns *p* > 0.05, * *p* < 0.05, ** *p* < 0.01, *** *p* < 0.001. (**B**) Two-dimensional scatter plots obtained from Annexin V-FITC/PI double-staining flow cytometry for apoptosis detection. Cell subpopulations were distinguished based on four quadrants in the scatter plot: dead cells/debris in the upper left quadrant, viable cells in the lower left quadrant, late apoptotic cells in the upper right quadrant, and early apoptotic cells in the lower right quadrant. (**C**) Statistical analysis of the percentages of early apoptotic cells and late apoptotic cells in each group; ns *p* > 0.05, * *p* < 0.05, ** *p* < 0.01, *** *p* < 0.001.

The CCK-8 assay results showed that TPL treatment reduced HTR-8/SVneo cell viability in a concentration-dependent manner after 48 h (Figure 1A). Cell viability dropped to 88.5% in the 5 nM group and further declined to 73.5% in the 10 nM group. When the concentration was increased to 15 nM and 20 nM, this cytotoxic effect became more pronounced.

### 3.2. TPL Concentration-Dependently Promotes Apoptosis

Flow cytometry results showed that after treating HTR-8/SVneo cells with TPL for 48 h, the total apoptosis rate, calculated as the sum of the percentages of early apoptotic cells (Q4 quadrant) and late apoptotic cells (Q2 quadrant), increased in a concentration-dependent manner (Figure 1B). Compared with the control group, no significant difference in apoptosis rate was observed in the 5 nM TPL group, while the apoptosis rate was significantly increased in the 10, 15, and 20 nM TPL groups (Figure 1C).

### 3.3. Prediction of Potential Targets of TPL

Potential targets of TPL were predicted using the Pharmmapper database, and a total of 252 candidate target proteins were obtained after screening combined with the UniProt human gene database. These targets were submitted to the DAVID database for GO functional annotation and KEGG pathway enrichment analysis (Figure S1D,E). The results showed that the target genes were mainly enriched in FoxO, p53, TNF, and MAPK signaling pathways, as well as terms closely related to cell survival and reproductive function, such as positive regulation of apoptotic process and mitochondrial outer membrane structure, providing directions for subsequent mechanistic studies.

### 3.4. Transcriptomic Analysis of TPL-Mediated Effects in HTR-8/SVneo Cells

**Figure 2:**
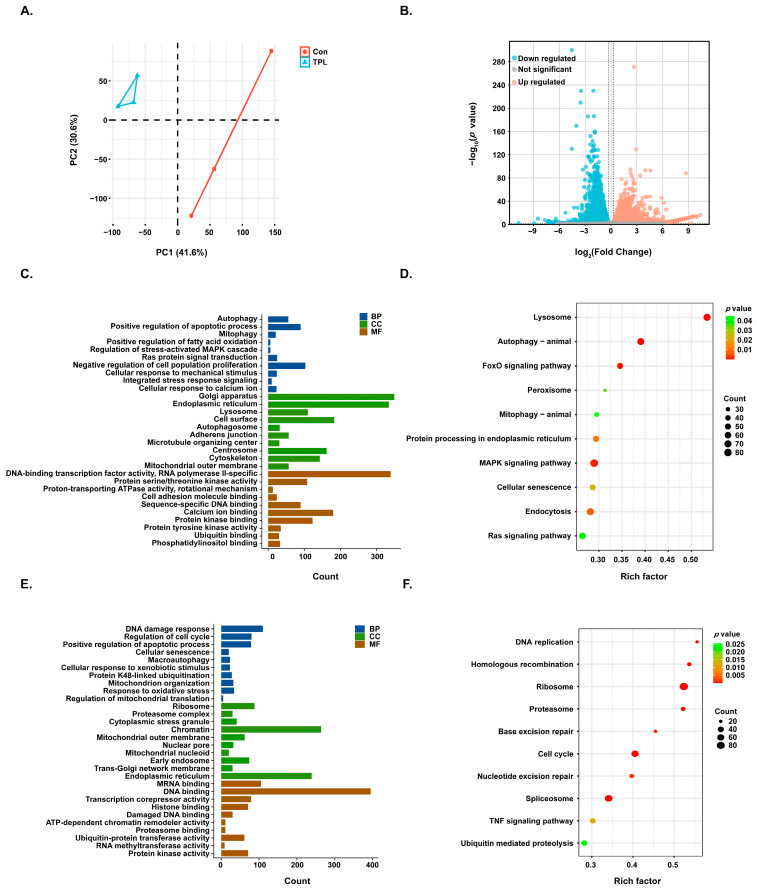
Bioinformatics analysis of mRNA-seq in HTR-8/SVneo cells treated with 0 and 15 nM TPL for 48 h. (**A**) PCA of mRNA expression in the control and TPL-treated groups. (**B**) Volcano plot showing DEGs following TPL treatment. Representative (**C**) GO and (**D**) KEGG enrichment analyses of upregulated DEGs. Representative (**E**) GO and (**F**) KEGG enrichment analyses of downregulated DEGs.

**Figure 3:**
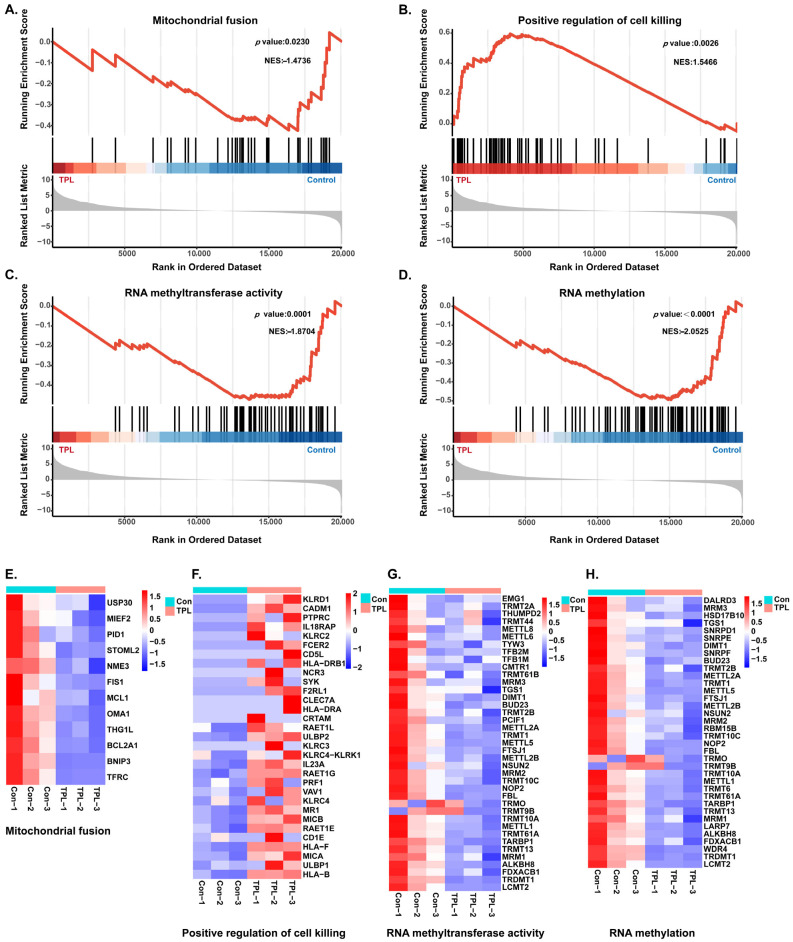
Identification of key biological processes and potential regulatory pathways involved in TPL treatment. (**A**–**D**) GSEA enrichment analysis was performed on 20,090 genes covered by mRNA-seq, identifying four gene sets closely associated with TPL treatment: (**A**) Mitochondrial fusion, (**B**) Positive regulation of cell killing, (**C**) RNA methyltransferase activity, and (**D**) RNA methylation. (**E**–**H**) Clustering heatmaps were generated using the core genes from the above four gene sets based on the mRNA-seq expression matrix: (**E**) Mitochondrial fusion, (**F**) Positive regulation of cell killing, (**G**) RNA methyltransferase activity, and (**H**) RNA methylation.

To clarify the transcriptional regulatory mechanism of TPL-induced reproductive toxicity, mRNA-seq analysis was performed on HTR-8/SVneo cells treated with 0 and 15 nM TPL. Principal component analysis (PCA) revealed that the control (Con) and TPL-treated groups (TPL) were completely separated along PC1 (contribution rate 41.6%), with a PC2 contribution rate of 30.6%, indicating significant differences in gene expression between the two groups and reliable experimental repeatability (Figure 2A). A total of 20,090 genes were covered in this sequencing. With |log_2_FC| ≥ log_2_(1.2) and *p*-value < 0.05 as the screening criteria for DEGs, 9023 DEGs were finally obtained, including 4890 upregulated genes and 4133 downregulated genes (Figure 2B). The upregulated and downregulated DEGs were submitted to the DAVID database for GO and KEGG enrichment analysis, respectively (Figure 2C–F). GO enrichment analysis showed that the DEGs were significantly enriched in key biological processes and molecular function terms such as RNA methyltransferase activity, regulation of cell cycle, cellular senescence, response to oxidative stress, and regulation of mitochondrial translation. KEGG enrichment results pointed to core pathways including peroxisome, endocytosis, DNA replication, mitophagy—animal, and Ras signaling pathway. To further identify the key biological processes and potential regulatory pathways of TPL action, GSEA enrichment analysis was performed on the 20,090 detected genes, and four terms closely related to TPL action were screened out, namely mitochondrial fusion, positive regulation of cell killing, RNA methyltransferase activity, and RNA methylation (Figure 3A–D). Among them, the TPL group showed a significant down-regulation trend in mitochondrial fusion, RNA methyltransferase activity, and RNA methylation functions, and a significant up-regulation trend in positive regulation of cell killing, suggesting that TPL may exert reproductive toxicity through the above pathways. The core genes in these four key terms were used to extract the original expression matrix of mRNA-seq and construct clustering heatmaps (Figure 3E–H). Visualization of the heatmaps showed that the expression of genes related to mitochondrial fusion, RNA methyltransferase activity, and RNA methylation was generally downregulated in the TPL group, while the expression of genes associated with positive regulation of cell killing was generally upregulated, which was highly consistent with the GSEA enrichment results, providing an important basis for further exploration of its core action pathways.

### 3.5. Effects of TPL on m^6^A Modification Patterns in HTR-8/SVneo Cells

**Figure 4:**
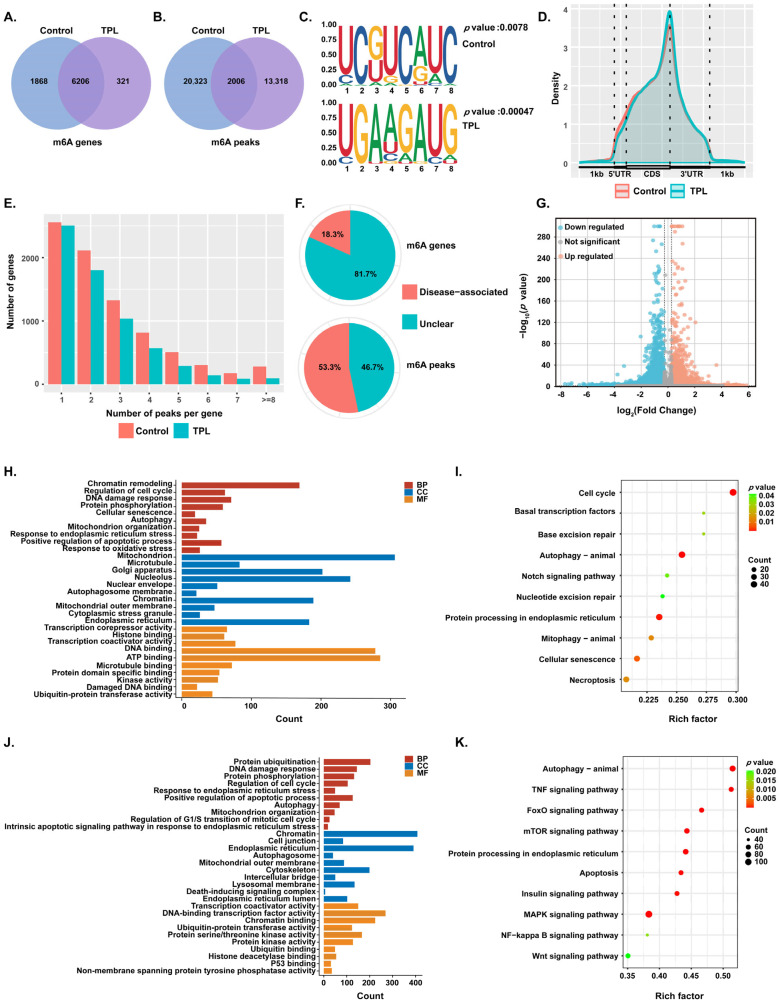
Analysis of m^6^A modification patterns in HTR-8/SVneo cells treated with TPL. (**A**) Venn diagram showing the overlap of m^6^A-modified genes between control and TPL-treated groups. (**B**) Venn diagram showing the overlap of m^6^A peaks between the two groups. (**C**) Enriched m^6^A motifs in control and TPL-treated groups. (**D**) Distribution of m^6^A peaks across transcripts. (**E**) Distribution of m^6^A peak numbers per gene. (**F**) Association analysis of m^6^A-modified genes and m^6^A peaks with diseases. (**G**) Volcano plot showing differentially m^6^A-modified gene regions following TPL treatment. Representative (**H**) GO and (**I**) KEGG enrichment analyses of upregulated differentially m^6^A-modified gene regions. Representative (**J**) GO and (**K**) KEGG enrichment analyses of downregulated differentially m^6^A-modified gene regions.

The above results suggested that RNA methyltransferase activity was downregulated after TPL treatment, indicating that TPL might exert its functions by influencing m^6^A modification, a key epitranscriptomic regulator of gene expression. Therefore, we performed MeRIP-seq to investigate whether TPL alters the RNA m^6^A methylation landscape. The results showed that 22,329 m^6^A peaks in 8074 m^6^A genes were detected in the control group, whereas only 15,324 m^6^A peaks in 6527 m^6^A-modified genes were identified in the TPL-treated group (Figure 4A,B). This marked reduction in both m^6^A-modified genes and modification sites aligns with the observed downregulation of RNA methyltransferase activity following TPL treatment. STREME analysis revealed that the conserved m^6^A methylation motif RRACH was present in both the control and TPL groups, suggesting that TPL did not alter the site specificity of m^6^A modification (Figure 4C). The distribution of m^6^A modifications along transcripts is not random, but mainly enriched in the CDS and 3′UTR (Figure 4D). Statistical analysis of m^6^A modification peaks showed that modifications with low peak numbers were dominant in both groups. In the control group, there were approximately 2500 genes with one peak and about 2000 genes with two peaks. The number of genes gradually decreased as the peak number increased. The TPL group exhibited the same distribution pattern. High-abundance modifications with more than four peaks existed only in very few transcripts (Figure 4E). RMDisease analysis revealed that 53.3% of m^6^A peaks and 18.3% of m^6^A-modified genes were associated with diseases, indicating that TPL-induced abnormal m^6^A modification may be closely related to reproductive injury (Figure 4F). According to the same screening criteria as mRNA-seq (|log_2_FC| ≥ log_2_(1.2) and *p*-value < 0.05), a total of 12,859 differential m^6^A-modified gene regions were identified after TPL treatment, including 3736 regions with upregulated m^6^A modification and 9123 regions with downregulated m^6^A modification (Figure 4G). GO and KEGG enrichment analyses were performed for both upregulated and downregulated different m^6^A-modified gene regions (Figure 4H–K). The results indicated that the differential m^6^A-modified genes were predominantly enriched in biological processes, including response to endoplasmic reticulum stress, damaged DNA binding, mitochondrion and autophagosome membrane, as well as pathways including protein processing in endoplasmic reticulum, apoptosis, Wnt signaling pathway, and base excision repair, which are highly associated with the toxic effects of TPL.

### 3.6. Integrated Analysis of mRNA-Seq and MeRIP-Seq

**Figure 5:**
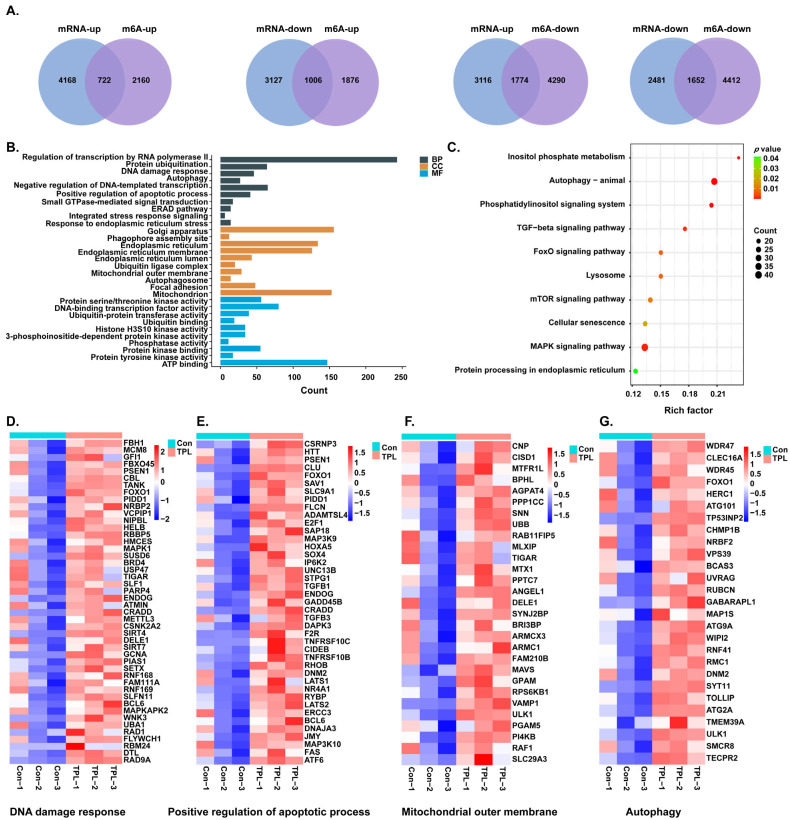
Integrated analysis of mRNA-seq and MeRIP-seq in TPL-treated HTR-8/SVneo cells. (**A**) Venn diagram showing the overlap between differentially m^6^A-modified gene regions and differentially expressed genes between the control and TPL-treated groups. Representative (**B**) GO and (**C**) KEGG enrichment analyses of genes with upregulated expression and decreased m^6^A modification. (**D**–**G**) Clustering heatmaps of four significantly enriched core pathways from the enrichment analysis: (**D**) DNA damage response, (**E**) Positive regulation of apoptotic process, (**F**) Mitochondrial outer membrane, and (**G**) Autophagy.

To elucidate the relationship between m^6^A modification and gene expression, an integrated analysis of MeRIP-seq and mRNA-seq data was performed to screen for genes exhibiting both differential expression and significantly altered m^6^A modification levels. The results showed that 722 genes were upregulated and 1006 genes were downregulated with enhanced m^6^A modification, while 1774 genes were upregulated and 1652 genes were downregulated with reduced m^6^A modification (Figure 5A). The overall pattern was characterized by a greater number of upregulated mRNA genes and a predominance of genes with decreased m^6^A modification, underscoring the potential regulatory role of m^6^A loss in driving mRNA accumulation. GO enrichment analysis of the downregulated mRNA gene set revealed significant enrichment of the term “RNA methyltransferase activity”, further supporting that TPL may suppress global m^6^A levels by inhibiting methyltransferase function (Figure 2E). Given that m^6^A modification often promotes mRNA degradation, the loss of m^6^A marks could impair the decay efficiency of target transcripts, thereby contributing to their elevated expression. These findings point to m^6^A reduction as a key mechanism underlying the upregulation of specific mRNAs in response to TPL treatment.

Therefore, we next investigated the functional implications of the genes whose up-regulation was associated with m^6^A loss. GO and KEGG enrichment analyses were performed on the 1774 overlapping genes exhibiting “mRNA up-regulation and m^6^A down-regulation” (Figure 5B,C). Four significantly enriched core pathways were identified. They are positive regulators of the apoptotic process, autophagy, DNA damage response, and mitochondrial outer membrane. The clustering heatmap results showed that all key genes in the above four pathways were consistently upregulated in the TPL-treated group, consistent with the enrichment analysis results (Figure 5D–G).

These results indicate that TPL induces global m^6^A modification loss by inhibiting RNA methyltransferase activity, thereby stabilizing the mRNAs of genes related to apoptosis, autophagy, DNA damage response, and mitochondrial outer membrane at the post-transcriptional level, and ultimately amplifying cellular damage signals.

### 3.7. Downregulation of Core Writer Regulatory Factors Mediates TPL-Induced m^6^A Loss

**Figure 6:**
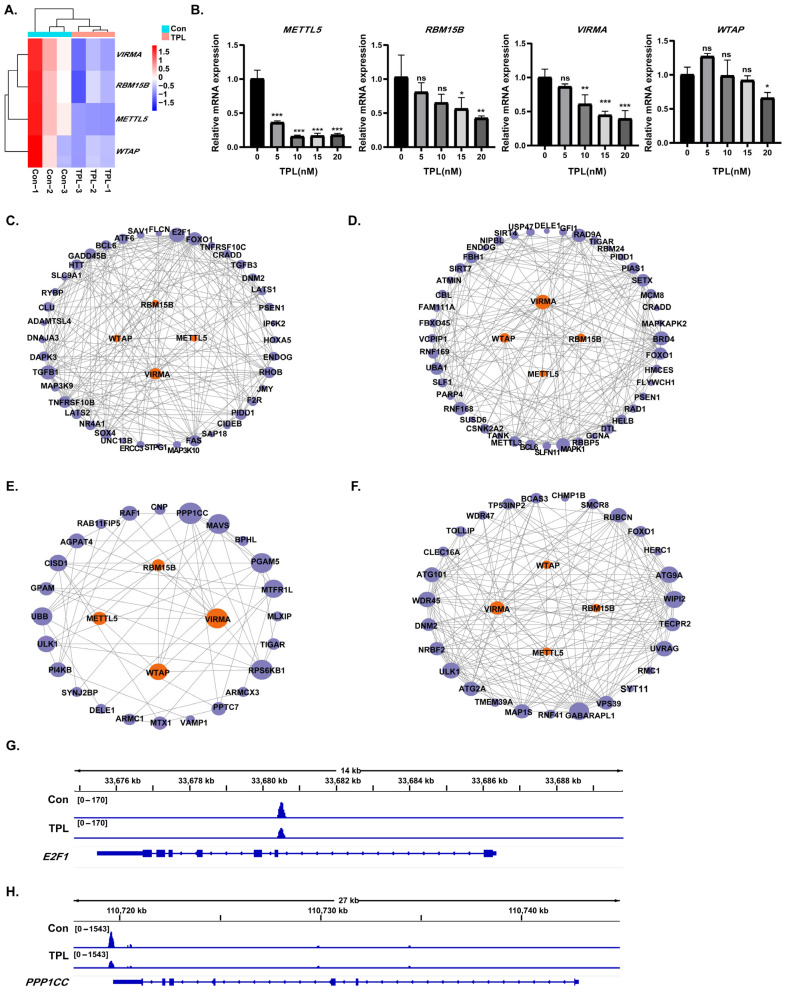
Differentially expressed potential m^6^A regulators involved in TPL treatment. (**A**) Clustering heatmap of four core writer genes. (**B**) mRNA expression levels of four writer genes detected by RT-qPCR; ns *p* > 0.05, * *p* < 0.05, ** *p* < 0.01, *** *p* < 0.001. (**C**–**F**) Protein–protein interaction networks between genes from four core pathways and the four writer genes: (**C**) Positive regulation of apoptotic process, (**D**) DNA damage response, (**E**) Mitochondrial outer membrane, and (**F**) Autophagy. (**G**,**H**) Visualization of m^6^A modification levels on mRNA transcripts of (**G**) *E2F1* and (**H**) *PPP1CC* genes by IGV.

mRNA expression changes in m^6^A regulators were analyzed by mRNA-seq to screen for potential regulators targeted by TPL. As shown in Table 2, the mRNA levels of *ALKBH5*, *YTHDF3*, *METTL3*, and *CBLL1* were markedly upregulated, whereas regulatory factors, including *HNRNPC*, *VIRMA*, *METTL5*, *WTAP*, *IGF2BP1*, *RBM15B*, *IGF2BP3*, *YTHDC1*, *IGF2BP2*, and *YTHDC2*, were obviously downregulated. Combined with the finding that “RNA methyltransferase activity” was significantly enriched in the “downregulated mRNA” gene set in the previous GO enrichment analysis, it was speculated that transcriptional inhibition of writer regulatory factors may be the upstream driving event of TPL-induced global m^6^A loss. Four core writers, *VIRMA*, *METTL5*, *WTAP*, and *RBM15B*, were selected from the downregulated regulatory factors for further analysis. The clustering heatmap showed that, compared to the control group, the expression levels of these four factors were significantly downregulated in the TPL group (Figure 6A). RT-qPCR analysis further confirmed that the mRNA expression levels of the four writers showed a gradient decrease with increasing TPL concentration, which was consistent with the sequencing results, verifying the reliability of the data (Figure 6B).

A PPI network was constructed between the genes involved in the four core pathways, positive regulation of apoptosis, DNA damage response, mitochondrial outer membrane, and autophagy, and the four writer factors (Figure 6C–F). The top three core genes with the highest degree values in each pathway were screened. They are *E2F1*, *TGFB1*, *FOXO1* (positive regulation of apoptotic process), *BRD4*, *RAD9A*, *MAPK1* (DNA damage response), *PPP1CC*, *MAVS*, *RPS6KB1* (mitochondrial outer membrane), and *GABARAPL1*, *WIPI2*, *ATG9A* (autophagy). *E2F1* and *PPP1CC*, which had the highest degree values in the pathways of positive regulation of the apoptotic process and mitochondrial outer membrane, respectively, were selected for visualization analysis of MeRIP-seq results using IGV. The results showed distinct m^6^A enrichment peaks in the exon regions of these two genes in the control group, whereas the peak heights were significantly reduced after TPL treatment, confirming that downregulation of writer factors directly leads to m^6^A modification loss in core pathway target genes (Figure 6G,H).

### 3.8. Stable Interaction Between TPL and Core Writer Factors

**Figure 7:**
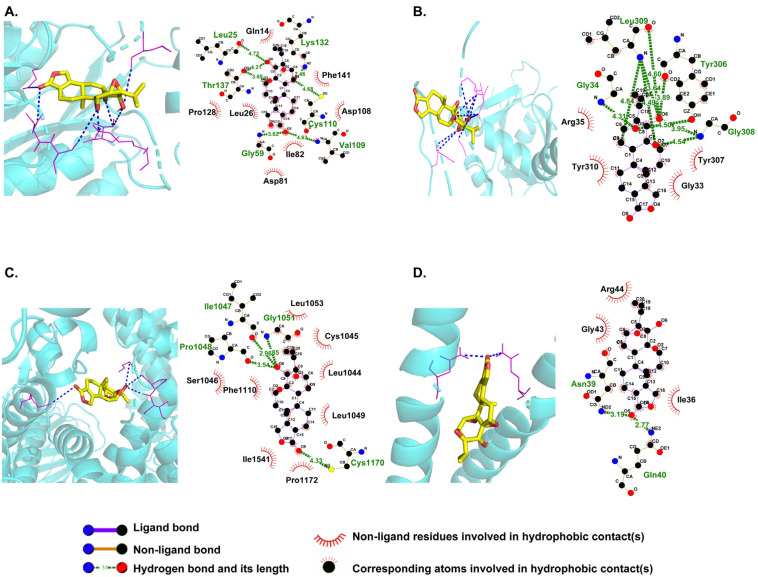
Molecular interactions between TPL and four core m^6^A regulators. (**A**) METTL5, (**B**) RBM15B, (**C**) VIRMA, and (**D**) WTAP.

To verify the direct interaction between TPL and writer factors, AutoDock molecular docking was performed between TPL and four core m^6^A regulators, VIRMA, METTL5, WTAP, and RBM15B, respectively. The basic protein information, docking binding energy, number of hydrogen bonds, and amino acid residues involved in hydrogen bond formation, as well as the 2D and 3D diagrams of the docking between TPL and each protein, are presented (Table 3; Figure 7A–D). Molecular docking revealed that the binding energies of TPL to VIRMA, METTL5, WTAP, and RBM15B were −6.23, −7.33, −5.30, and −5.61 kcal/mol, respectively, all of which were lower than −1.2 kcal/mol, indicating that TPL could form stable complexes with these four regulatory factors. These results are consistent with previously reported mechanisms, where small molecules bind stably to target proteins through hydrogen bonds and regulate their functions [36]. These findings provide direct evidence for clarifying the molecular mechanism by which TPL regulates m^6^A modification.

## 4. Discussion

TPL, as the core active component of TwHF, exhibits a variety of pharmacological activities, including anti-inflammatory, immunomodulatory, anti-proliferative, and pro-apoptotic effects [37]. It shows broad prospects in tumor therapy. TPL and its derivatives can inhibit tumor growth and metastasis. They enhance drug sensitivity in drug-resistant cancers. When combined with existing anti-tumor drugs, they exert synergistic anti-tumor effects on gynecological cancers and breast cancer [38]. Meanwhile, TPL can inhibit the polarization of M2-type tumor-associated macrophages by targeting the PI3K/AKT/NF-κB signaling pathway, thereby inhibiting invasion and migration of drug-resistant ovarian cancer, highlighting its potential value in inhibiting cancers of the female reproductive system [39]. However, with the deepening of research on the clinical application of TPL, its toxic effects have become increasingly prominent. Previous studies have demonstrated that TPL induces cytotoxicity via multiple mechanisms. These mechanisms include membrane injury, mitochondrial damage, metabolic dysfunction, endoplasmic reticulum stress, oxidative stress, apoptosis, and autophagy, and such effects further lead to multi-organ damage in animals and humans [40]. In the liver, TPL triggers ferroptosis in hepatocytes, as evidenced by iron accumulation and lipid peroxidation, and thus causes liver injury [41]. In the kidney, administration of TPL at 100–400 μg/kg changes the localization of ZO-1 in proximal tubular epithelial cells and elevates paracellular permeability in NRK-52E cell monolayers. These alterations disrupt renal homeostasis and contribute to proximal tubular injury [42]. In the heart, the p53 protein serves a key function in cardiomyocyte apoptosis. TPL induces cardiomyocyte apoptosis by activating the expression of p53 and its target genes and facilitating the translocation of p53 from the cytoplasm to the nucleus [43]. In the reproductive system, the toxic effect of TPL is more significant. In male mice, TPL can increase macrophages and inflammatory responses in the testes, enhance reactive oxygen species (ROS) signaling in somatic cells, especially Leydig cells and Sertoli cells, and induce the downregulation of pathways related to sperm cell development [44]. In female mice, TPL affects mitochondrial function and increases mitochondrial outer membrane permeability, leading to the release of mitochondrial DNA into the cytoplasm and thereby activating the cGAS-STING pathway, which causes ovarian inflammation and decreased oocyte quality [45]. In in vitro cell experiments, researchers found that after culturing the human choriocarcinoma cell line JEG-3, TPL can interfere with the normal synthesis of sex steroid hormones by inhibiting aromatase, a key rate-limiting enzyme in the conversion of androgens to estrogens [46]. However, studies on the specific molecular mechanism of TPL-induced reproductive toxicity are quite limited, which severely restricts the safe clinical medication and dosage form optimization of TwHF.

In this study, the HTR-8/SVneo cells were used as a model. These cells are key functional cells for placentation and embryonic development, and can mimic the core biological behaviors of primary trophoblast cells, such as invasion and immune regulation. Their functional status is directly associated with pregnancy outcomes and reproductive health [47]. Accordingly, this study performed an integrated multi-dimensional correlation analysis at the epitranscriptomic level by combining MeRIP-seq and mRNA-seq, aiming to systematically elucidate the molecular mechanism underlying TPL-induced reproductive toxicity.

CCK-8 assay results showed that TPL treatment for 48 h reduced the cell viability of HTR-8/SVneo cells in a concentration-dependent manner. Meanwhile, flow cytometry results demonstrated that the total apoptosis rate increased in a concentration-dependent manner. These results confirm that the toxic effect of TPL on these cells exhibits clear concentration dependence. We compared our results with those of a study on TPL-induced toxicity in cholangiocarcinoma cells [48]. That study confirmed that TPL toxicity is time-dependent, as the reduction in cell viability at 48 h was significantly stronger than that at 24 h, which supports our choice of 48 h treatment for HTR-8 cells. Regarding the CCK-8 assay, the findings of that study are consistent with ours. TPL reduced cell viability in a concentration-dependent manner. That study reported a Half-Maximal Inhibitory Concentration (IC_50_) of 12.5–25 nM in cholangiocarcinoma cells after 48 h of TPL treatment, whereas our IC_50_ in HTR-8/SVneo cells was 15–20 nM. These values are very close, indicating that our selected drug concentrations are reasonable. Regarding apoptosis detection by flow cytometry, the results of that study are also consistent with ours. The apoptosis rate increased with increasing TPL concentration. In summary, our experimental conditions are justified, and our results are consistent with the concentration-dependent toxicity profile of TPL reported in the literature.

mRNA-seq analysis showed that 9023 DEGs were identified after TPL treatment, among which downregulated genes were significantly enriched in the term “RNA methyltransferase activity”. GSEA enrichment results further indicated that TPL could inhibit functions related to RNA methylation and promote functions related to cell killing, preliminarily suggesting the central role of abnormal m^6^A modification in TPL-induced toxicity. The MeRIP-seq assay clarified the regulatory characteristics of TPL on m^6^A modification. After TPL treatment, both the total number of m^6^A-modified genes and the overall number of modification peaks were significantly decreased. Moreover, m^6^A modifications were predominantly enriched in the CDS and 3′UTR regions of transcripts, while the modification specificity of the RRACH conserved motif remained unchanged. In addition, 18.3% of m^6^A-modified genes were associated with diseases, further confirming that TPL-induced m^6^A abnormality may be the key epitranscriptomic basis for its reproductive toxicity.

To deeply explore the reproductive toxicity mechanism of TPL, this study identified 1774 key genes with mRNA upregulation and m^6^A downregulation by integrating the results of mRNA-seq and MeRIP-seq. These genes were significantly enriched in four core pathways. The pathways included positive regulation of the apoptotic process, autophagy, DNA damage response, and the mitochondrial outer membrane. Among them, DNA damage response is an important defense mechanism for cells against genetic material damage, and its core ATM/ATR-γH2AX-p53 pathway participates in signal transduction and repair regulation after injury [49]. Changes in mitochondrial outer membrane permeability are key events in mitochondrial dysfunction and the initiation of cellular apoptosis, which are precisely regulated by Bcl-2 family proteins [50]. Autophagy plays a dual role in maintaining cellular homeostasis. Moderate autophagy helps remove damaged components, while excessive autophagy may lead to cell death [51]. As the main form of programmed cell death, apoptosis execution relies on the activation of the caspase cascade [52]. In this study, genes related to these pathways all showed m^6^A downregulation and mRNA upregulation after TPL treatment, suggesting that TPL may interfere with the normal functions of these pathways through epitranscriptomic regulation and synergistically aggravate trophoblast cell injury. Consistent with previous studies, epigenetic regulation is a central part of cellular functional pathway regulation, which directly affects physiological functions and stress responses by targeting the expression of specific pathway genes [53].

To clarify the upstream mechanism underlying aberrant m^6^A modification, this study focused on the expression changes in m^6^A regulators and found that four core writer factors (*VIRMA*, *METTL5*, *WTAP*, and *RBM15B*) were downregulated in a concentration-dependent manner, consistent with the inhibitory trend of “RNA methyltransferase activity”. IGV visualization confirmed that the downregulation of these writer factors could directly reduce the m^6^A modification levels of *E2F1* (a core gene in the apoptotic pathway) and *PPP1CC* (a core gene in the mitochondrial outer membrane pathway). Molecular docking assays further provided direct evidence. The binding energies between TPL and these four writer factors were all less than −1.2 kcal/mol, indicating that stable binding could be formed between TPL and these factors.

Based on the above results, this study proposes the molecular mechanism underlying TPL-induced reproductive toxicity. TPL directly binds to writer factors such as VIRMA, METTL5, WTAP, and RBM15B, thereby inhibiting their expression activity and leading to a global reduction in m^6^A modification levels. Loss of m^6^A modification on core pathway target genes (e.g., *E2F1* and *PPP1CC*) reduces their mRNA degradation and upregulates their expression. This further activates the DNA damage response, mitochondrial outer membrane, autophagy, and positive regulation of apoptotic process pathways, ultimately inducing trophoblast cell injury and triggering reproductive toxicity. Unlike previous studies on TPL toxicity that have largely focused on downstream effects such as oxidative stress, this study proposes that aberrant m^6^A modification acts as the core upstream initiating event underlying these downstream processes, filling a research gap in the epitranscriptional regulatory mechanisms of TPL-induced reproductive toxicity [40]. Previous studies have demonstrated that *WTAP* plays a critical role in trophoblast cell proliferation, placental development, and perinatal growth [54]. Notably, this study directly links m^6^A writers (*VIRMA*, *METTL5*, *WTAP*, and *RBM15B*) to TPL-mediated reproductive toxicity, verifying them as direct targets of TPL. This breaks the limitation that previous research on TPL targets has been predominantly concentrated on tumor-related pathways, enriches the target spectrum of TPL toxicity, and provides novel molecular targets for the subsequent targeted intervention of TPL-induced reproductive toxicity [55].

However, this study still has certain limitations. As a triepoxide compound, TPL can react with a large number of intracellular nucleophilic substances, including proteins, nucleotides, and peptides, to form covalent adducts, thereby interfering with epitranscriptomic modifications and gene expression through various pathways such as electrophilic damage and oxidative stress. In the future, we will further investigate the covalent binding profile of TPL and its indirect effects on the m^6^A regulatory network to more comprehensively elucidate the molecular mechanism of its reproductive toxicity.

From the epitranscriptomic perspective, this study explored the reproductive toxicity mechanism of TPL and found that abnormal m^6^A modification may be the core regulatory event, providing new insights and experimental evidence for mechanistic research in this field. By employing a research strategy that integrates multi-omics analysis, molecular docking and experimental verification, this study offers a referable paradigm for subsequent research on the toxicity mechanism of active ingredients in traditional Chinese medicine. Furthermore, this study identified target genes, including *E2F1* and *PPP1CC*, as potential biomarkers of TPL-induced reproductive toxicity, offering target support for the later development of attenuated preparations and the establishment of safe medication standards.

## 5. Conclusions

This study confirms that TPL can directly bind to m^6^A writer regulatory factors such as VIRMA, METTL5, WTAP, and RBM15B and inhibit their expression, downregulate the m^6^A modification levels of target genes in pathways including positive regulation of apoptosis, autophagy, DNA damage response, and mitochondrial outer membrane, promote the stability of mRNA, and upregulate the expression of these genes. These events ultimately activate cell death signaling pathways, induce trophoblast cell injury, and trigger reproductive toxicity. These findings provide a new epitranscriptomic perspective for elucidating the mechanism underlying TPL-induced reproductive injury, reveal the crucial role of RNA methylation modification in the regulation of traditional Chinese medicine toxicity, and open up new research directions for the in-depth mechanistic dissection of traditional Chinese medicine toxicity.

## Figures and Tables

**Table 1:** Primers sequences used for RT-qPCR analyses.

Gene	Forward Primer (5′-3′)	Reverse Primer (5′-3′)
*VIRMA*	CGAGCGCTGAGCAAAGTTC	CAGCCTCTTAGCACCAGACC
*METTL5*	GGGTTAGCCGGGAGATCCT	ATCCAACACACAACCCTGCT
*WTAP*	GCTTCTGCCTGGAGAGGATT	TGCAGACTCCTGCTGTTGTT
*RBM15B*	CCCCTGGAGAGTTTTAGCCG	TCCACCTTTTCACTCACCCG

**Table 2:** mRNA expression levels of m^6^A regulators in TPL-treated HTR-8/SVneo cells.

Genes	Regulation	Base Mean	log2Fold Change	*p*-Value
*HNRNPC*	reader	58,416.35	−1.5085	6.18 × 10^−52^
*VIRMA*	writer	7187.03	−1.3333	1.52 × 10^−13^
*METTL5*	writer	2473.9	−0.7419	1.46 × 10^−12^
*WTAP*	writer	4223.12	−0.3441	1.96 × 10^−6^
*IGF2BP1*	reader	7619.98	−0.7686	2.53 × 10^−5^
*RBM15B*	writer	3766.17	−0.9475	2.60 × 10^−5^
*IGF2BP3*	reader	1712.64	−0.7767	3.28 × 10^−5^
*YTHDC1*	reader	16,337.26	−0.5729	3.39 × 10^−5^
*IGF2BP2*	reader	12,856.88	−0.3273	2.60 × 10^−4^
*ALKBH5*	eraser	18,918.31	0.4496	3.36 × 10^−4^
*YTHDF3*	reader	980.51	1.3334	7.30 × 10^−4^
*METTL3*	writer	2206.28	0.3842	9.02 × 10^−4^
*YTHDC2*	reader	1682.96	−0.3132	3.01 × 10^−3^
*CBLL1*	writer	1846.42	0.3985	3.21 × 10^−3^
*ZC3H13*	writer	42,261.46	−0.2529	6.68 × 10^−3^
*YTHDF1*	reader	3459.87	0.2628	8.30 × 10^−3^
*METTL14*	writer	4981.41	−0.2119	3.30 × 10^−2^
*YTHDF2*	reader	1683.61	−0.1843	1.83 × 10^−1^
*HNRNPA2B1*	reader	169,805.13	−0.0982	2.09 × 10^−1^
*RBM15*	writer	1187.04	0.4166	2.61 × 10^−1^
*FTO*	eraser	1624.24	0.1676	3.12 × 10^−1^
*FMR1*	reader	4457.33	−0.0487	6.28 × 10^−1^

**Table 3:** Molecular interactions between TPL and m^6^A regulators.

Protein Name	PDB ID or AlphaFold ID	Binding Energy (kcal/moL)	H-Bond Number (≤5 Å)	Residues Involved in H-Bond Formation
VIRMA	AF-Q69YN4-F1-model_v4	−6.23	4	Pro1048, Ile1047, Gly1051, Cys1170
METTL5	6h2v	−7.33	7	Leu25, Thr137 (2H-bonds), Gly59, Val109, Cys110, Lys132
WTAP	7yfj	−5.30	2	Asn39, Gln40
RBM15B	AF-Q8NDT2-F1-model_v4	−5.61	9	Gly34, Leu309 (4H-bonds), Tyr306 (2H-bonds), Gly308 (2H-bonds)

## Data Availability

The mRNA-seq and MeRIP-seq data generated in this study have been deposited in the NCBI Gene Expression Omnibus (GEO, https://www.ncbi.nlm.nih.gov/geo/, accessed on 23 February 2026) under accession numbers GSE320198 for the experimental group and GSE269246 for the control group.

## Supplementary Materials

The following supporting information can be downloaded at: https://www.mdpi.com/article/10.3390/toxics14040334/s1. Figure S1: Chemical characterization of TPL. (A) Chemical structure of TPL. (B) 1H NMR spectrum of TPL. (C) Purity analysis of TPL by HPLC. Representative (D) GO(Gene Ontology) terms and (E) KEGG(Kyoto Encyclopedia of Genes and Genomes) pathway enrichment analysis of potential TPL target proteins identified by the PharmMapper database.

## Abbreviations

The following abbreviations are used in this manuscript:

TPL	Triptolide
TwHF	*Tripterygium wilfordii* Hook F
m^6^A	N^6^-methyladenosine
CCK-8	Cell Counting Kit-8
DEGs	Differentially Expressed Genes
RT-qPCR	Real-Time quantitative PCR
NMR	Nuclear Magnetic Resonance
HPLC	High-Performance Liquid Chromatography
GO	Gene Ontology
KEGG	Kyoto Encyclopedia of Genes and Genomes
PPI	Protein–Protein Interaction
IGV	Integrative Genomics Viewer
ANOVA	Analysis of Variance
SEM	Standard Error of the Mean
PCA	Principal Component Analysis
ROS	Reactive Oxygen Species

## References

[1] Jiang M., Xie Y., Wang P., Du M., Wang Y., Yan S. (2024). Research Progress of Triptolide Against Fibrosis. Drug Des. Dev. Ther..

[2] Piekarz J., Picheta N., Pobideł J., Daniłowska K., Gil-Kulik P. (2025). Phytotherapy as an adjunct to the treatment of rheumatoid arthritis—A systematic review of clinical trials. Phytomedicine.

[3] Shu H., Chen X.Y., Zhao J., Li P., Sun Z. (2025). Efficacy and safety of *Tripterygium wilfordii* glycosides tablets combined with Western medicine for patients with rheumatic immune diseases. World J. Clin. Cases.

[4] Zhou Y., Liang H., Yan J., He X., Pan L., Li X., Chen X., Chen X., Yang A., Huang Q. (2022). Effectiveness and safety of tripterygium glycosides tablet for lupus nephritis: A systematic review and Meta-analysis. J. Tradit. Chin. Med..

[5] Chan W.Y., Ng T.B. (1995). Adverse effect of *Tripterygium wilfordii* extract on mouse embryonic development. Contraception.

[6] Zhang H.R., Li Y.P., Shi Z.J., Liang Q.Q., Chen S.Y., You Y.P., Yuan T., Xu R., Xu L.H., Ouyang D.Y. (2023). Triptolide induces PANoptosis in macrophages and causes organ injury in mice. Apoptosis.

[7] Zheng Z., Yan G., Xi N., Xu X., Zeng Q., Wu Y., Zheng Y., Zhang G., Wang X. (2023). Triptolide Induces Apoptosis and Autophagy in Cutaneous Squamous Cell Carcinoma via Akt/mTOR Pathway. Anticancer Agents Med. Chem..

[8] Zhang H., Guo M., Zhang P., Mu B., Bai Z., Li L., Yu J. (2024). Triptolide promotes nerve repair after cerebral ischemia reperfusion injury by regulating the NogoA/NgR/ROCK pathway. Folia Neuropathol..

[9] Luo Z., Liao T., Zhang Y., Zheng H., Sun Q., Han F., Yang Z., Sun Q. (2020). Triptolide Attenuates Transplant Vasculopathy Through Multiple Pathways. Front. Immunol..

[10] Xu Y., Fan Y.F., Zhao Y., Lin N. (2019). Overview of reproductive toxicity studies on *Tripterygium wilfordii* in recent 40 years. Zhongguo Zhong Yao Za Zhi.

[11] Izadpanah A., Rappaport J., Datta P.K. (2022). Epitranscriptomics of SARS-CoV-2 Infection. Front. Cell Dev. Biol..

[12] Minervini C.F., Parciante E., Impera L., Anelli L., Zagaria A., Specchia G., Musto P., Albano F. (2020). Epitranscriptomics in Normal and Malignant Hematopoiesis. Int. J. Mol. Sci..

[13] Meyer K.D., Saletore Y., Zumbo P., Elemento O., Mason C.E., Jaffrey S.R. (2012). Comprehensive analysis of mRNA methylation reveals enrichment in 3′ UTRs and near stop codons. Cell.

[14] Huang D., Meng J., Chen K. (2024). AI techniques have facilitated the understanding of epitranscriptome distribution. Cell Genom..

[15] Yang Y., Hsu P.J., Chen Y.-S., Yang Y.-G. (2018). Dynamic transcriptomic m6A decoration: Writers, erasers, readers and functions in RNA metabolism. Cell Res..

[16] Zhao B.S., Roundtree I.A., He C. (2017). Post-transcriptional gene regulation by mRNA modifications. Nat. Rev. Mol. Cell Biol..

[17] Zhang Y., Wu Y., Ma J., Wu Y., Li L., Wang H., Jia G., Rigden D.J., Meng J., Huang D. (2025). DirectRM: Integrated detection of landscape and crosstalk between multiple RNA modifications using direct RNA sequencing. Nat. Commun..

[18] Gauster M., Moser G., Wernitznig S., Kupper N., Huppertz B. (2022). Early human trophoblast development: From morphology to function. Cell. Mol. Life Sci..

[19] Takei A., Nagashima G., Suzuki R., Hokaku H., Takahashi M., Miyo T., Asai J., Sanada Y., Fujimoto T. (1997). Meningoencephalocele associated with *Tripterygium wilfordii* treatment. Pediatr. Neurosurg..

[20] Wu Q., Chen M., Lin Y., Zhang J., Gao X., Wu Y., Wu C., Wen J., Li J., Li C. (2024). Multiomics profiling uncovers curdione-induced reproductive toxicity in HTR-8/SVneo cells. Heliyon.

[21] Lin B., Zhang J., Chen M., Gao X., Wen J., Tian K., Wu Y., Chen Z., Yang Q., Zhu A. (2024). Comprehensive Profiling of Transcriptome and m6A Epitranscriptome Uncovers the Neurotoxic Effects of Yunaconitine on HT22 Cells. Evol. Bioinform. Online.

[22] Chen H., Kapidzic M., Gantar D., Aksel S., Levan J., Abrahamsson D.P., Jigmeddagva U., Basrai S., San A., Gaw S.L. (2023). Perfluorooctanoic acid induces transcriptomic alterations in second trimester human cytotrophoblasts. Toxicol. Sci..

[23] Lapehn S., Houghtaling S., Ahuna K., Kadam L., MacDonald J.W., Bammler T.K., LeWinn K.Z., Myatt L., Sathyanarayana S., Paquette A.G. (2023). Mono(2-ethylhexyl) phthalate induces transcriptomic changes in placental cells based on concentration, fetal sex, and trophoblast cell type. Arch. Toxicol..

[24] Kim D., Paggi J.M., Park C., Bennett C., Salzberg S.L. (2019). Graph-based genome alignment and genotyping with HISAT2 and HISAT-genotype. Nat. Biotechnol..

[25] Meng J., Lu Z., Liu H., Zhang L., Zhang S., Chen Y., Rao M.K., Huang Y. (2014). A protocol for RNA methylation differential analysis with MeRIP-Seq data and exomePeak R/Bioconductor package. Methods.

[26] Bailey T.L. (2021). STREME: Accurate and versatile sequence motif discovery. Bioinformatics.

[27] Pertea M., Kim D., Pertea G.M., Leek J.T., Salzberg S.L. (2016). Transcript-level expression analysis of RNA-seq experiments with HISAT, StringTie and Ballgown. Nat. Protoc..

[28] Liu S., Wang Z., Zhu R., Wang F., Cheng Y., Liu Y. (2021). Three Differential Expression Analysis Methods for RNA Sequencing: Limma, EdgeR, DESeq2. J. Vis. Exp..

[29] Dennis G., Sherman B.T., Hosack D.A., Yang J., Gao W., Lane H.C., Lempicki R.A. (2003). DAVID: Database for Annotation, Visualization, and Integrated Discovery. Genome Biol..

[30] Milano M., Zucco C., Settino M., Cannataro M. (2022). An Extensive Assessment of Network Embedding in PPI Network Alignment. Entropy.

[31] Shannon P., Markiel A., Ozier O., Baliga N.S., Wang J.T., Ramage D., Amin N., Schwikowski B., Ideker T. (2003). Cytoscape: A software environment for integrated models of biomolecular interaction networks. Genome Res..

[32] Chen K., Song B., Tang Y., Wei Z., Xu Q., Su J., de Magalhães J.P., Rigden D.J., Meng J. (2021). RMDisease: A database of genetic variants that affect RNA modifications, with implications for epitranscriptome pathogenesis. Nucleic Acids Res..

[33] Bao X., Zhang Y., Li H., Teng Y., Ma L., Chen Z., Luo X., Zheng J., Zhao A., Ren J. (2023). RM2Target: A comprehensive database for targets of writers, erasers and readers of RNA modifications. Nucleic Acids Res..

[34] Livak K.J., Schmittgen T.D. (2001). Analysis of relative gene expression data using real-time quantitative PCR and the 2^−ΔΔCT^ Method. Methods.

[35] Tian J., Zhuang Y., Liu Y., Zheng Y., Liu X., Lin S., Zheng C., Wu Z. (2025). ROS-Mediated Unfolded Protein Response Activation Drives Hepatocyte Apoptosis in Mesaconitine-Induced Liver Injury. Toxics.

[36] Zheng Y., Liu Y., Tian J., Liu S., Ma G., Xie Y., Zheng C., Wu Z. (2025). Ochratoxin A induces immunotoxicity by targeting Annexin A1 mediated neutrophil apoptosis in zebrafish. Front. Immunol..

[37] Liu Q. (2011). Triptolide and its expanding multiple pharmacological functions. Int. Immunopharmacol..

[38] Li M., Li J., Tang Q., Zhu Y. (2024). Potential antitumor activity of triptolide and its derivatives: Focused on gynecological and breast cancers. Biomed. Pharmacother..

[39] Le F., Yang L., Han Y., Zhong Y., Zhan F., Feng Y., Hu H., Chen T., Tan B. (2021). TPL Inhibits the Invasion and Migration of Drug-Resistant Ovarian Cancer by Targeting the PI3K/AKT/NF-κB-Signaling Pathway to Inhibit the Polarization of M2 TAMs. Front. Oncol..

[40] Xi C., Peng S., Wu Z., Zhou Q., Zhou J. (2017). Toxicity of triptolide and the molecular mechanisms involved. Biomed. Pharmacother..

[41] Guo L., Yang Y., Ma J., Xiao M., Cao R., Xi Y., Li T., Huang T., Yan M. (2024). Triptolide induces hepatotoxicity by promoting ferroptosis through Nrf2 degradation. Cell Biol. Toxicol..

[42] Sun L., Li H., Huang X., Wang T., Zhang S., Yang J., Huang S., Mei H., Jiang Z., Zhang L. (2013). Triptolide alters barrier function in renal proximal tubular cells in rats. Toxicol. Lett..

[43] Xi Y., Wang W., Wang L., Pan J., Cheng Y., Shen F., Huang Z. (2018). Triptolide induces p53-dependent cardiotoxicity through mitochondrial membrane permeabilization in cardiomyocytes. Toxicol. Appl. Pharmacol..

[44] Zhang W., Xia S., Ou J., Cao M., Cheng G., Li Z., Wang J., Yang C. (2023). A single-cell landscape of triptolide-associated testicular toxicity in mice. J. Pharm. Anal..

[45] Cheng S.Y., Yang Y.F., Wang Y.L., Yue Z.P., Chen Y.Z., Wang W.K., Xu Z.R., Li L.F., Shen H., Qi Z.M. (2025). Triptolide exposure triggers ovarian inflammation by activating cGAS-STING pathway and decrease oocyte quality in mouse. Food Chem. Toxicol..

[46] Zhang J., Jiang Z., Zhang L. (2011). Effect of triptolide on aromatase activity in human placental microsomes and human placental JEG-3 cells. Arzneimittelforschung.

[47] Wang S., Qian J., Sun F., Li M., Ye J., Li M., Du M., Li D. (2019). Bidirectional regulation between 1st trimester HTR8/SVneo trophoblast cells and in vitro differentiated Th17/Treg cells suggest a fetal-maternal regulatory loop in human pregnancy. Am. J. Reprod. Immunol..

[48] Ding X., Zhang B., Pei Q., Pan J., Huang S., Yang Y., Zhu Z., Lv Y., Zou X. (2014). Triptolide induces apoptotic cell death of human cholangiocarcinoma cells through inhibition of myeloid cell leukemia-1. BMC Cancer.

[49] Ciccia A., Elledge S.J. (2010). The DNA damage response: Making it safe to play with knives. Mol. Cell.

[50] Popgeorgiev N., Gil C., Berthenet K., Bertolin G., Ichim G. (2024). Shedding light on mitochondrial outer-membrane permeabilization and membrane potential: State of the art methods and biosensors. Semin. Cell Dev. Biol..

[51] Liu S., Yao S., Yang H., Liu S., Wang Y. (2023). Autophagy: Regulator of cell death. Cell Death Dis..

[52] Newton K., Strasser A., Kayagaki N., Dixit V.M. (2024). Cell death. Cell.

[53] Lin W., Shi Y., Tian J., Liu X., Weng F., Wu Z. (2025). Kdm7aa Orchestrates an Immunomodulatory Cardiomyocyte Program to Enable Zebrafish Heart Regeneration. Int. J. Mol. Sci..

[54] Wu S., Liu K., Cui Y., Zhou B., Zhao H., Xiao X., Zhou Q., Ma D., Li X. (2024). N6-methyladenosine dynamics in placental development and trophoblast functions, and its potential role in placental diseases. Biochim. Biophys. Acta Mol. Basis Dis..

[55] Jiang H.Y., Bao Y.N., Lin F.M., Jin Y. (2021). Triptolide regulates oxidative stress and inflammation leading to hepatotoxicity via inducing CYP2E1. Hum. Exp. Toxicol..

