# Supplementary material for: Reprogramming of the m6A Epitranscriptome Drives Triptolide-Induced Reproductive Toxicity in HTR-8/SVneo Cells
Source: Toxics. 2026 Apr 16;14(4):334. doi: 10.3390/toxics14040334 (PMC13119796; doi:10.3390/toxics14040334)
Supplement: Supplementary file 1 [file toxics-14-00334-s001.zip › toxics-4217377-supplementary.pdf]

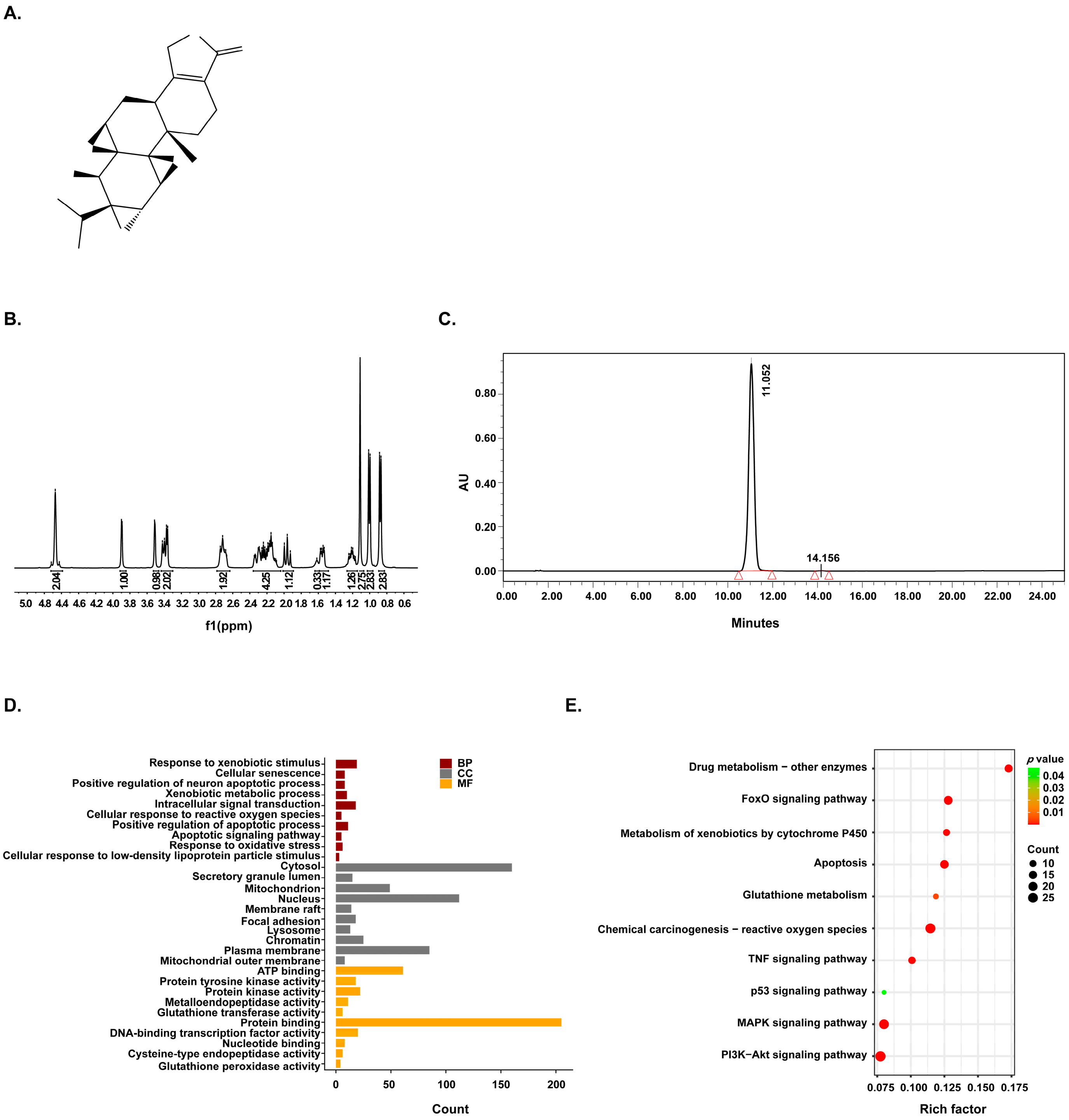

Figure S1. Chemical characterization of TPL. (A) Chemical structure of TPL. (B) ^{1}H NMR spectrum of TPL. (C) Purity analysis of TPL by HPLC. Representative (D) GO(Gene Ontology) terms and (E) KEGG(Kyoto Encyclopedia of Genes and Genomes) pathway enrichment analysis of potential TPL target proteins identified by the PharmMapper database.
